# A Pig Model of the Preterm Neonate: Anthropometric and Physiological Characteristics

**DOI:** 10.1371/journal.pone.0068763

**Published:** 2013-07-09

**Authors:** Yvonne A. Eiby, Layne L. Wright, Viskasari P. Kalanjati, Stephanie M. Miller, Stella T. Bjorkman, Helen L. Keates, Eugenie R. Lumbers, Paul B. Colditz, Barbara E. Lingwood

**Affiliations:** 1 UQ Centre for Clinical Research, The University of Queensland, Brisbane, Queensland, Australia; 2 School of Veterinary Science, The University of Queensland, Brisbane, Queensland, Australia; 3 School of Biomedical Sciences and Pharmacy and the Hunter Medical Research Institute, The University of Newcastle, Newcastle, New South Wales, Australia; Université de Montréal, Canada

## Abstract

**Background:**

Large animal models are an essential tool in the development of rationally-based new clinical therapies for preterm infants. We provide a description of the newborn pig as a model of the preterm neonate in terms of growth parameters, physiology and the requirement for intensive care over a range of gestational ages.

**Methods:**

Twenty-nine litters of piglets (n = 298) were delivered by caesarean section at six timepoints during gestation from 91d to 113d (term = 115d). Two groups, at 91 and 97d gestation, also received maternal glucocorticoid treatment. At four of these timepoints, piglets (n = 79) were ventilated, sedated and monitored using standard neonatal intensive care techniques for up to 8 h in various experimental protocols.

**Results:**

Body weight increased from mean 697 g (SD 193) at 91d gestation to 1331 g (SD 368) at 113d gestation. Piglets delivered at 97d gestation were able to be resuscitated and kept alive for at least 8 h on respiratory support after surfactant administration. Maternal glucocorticoid treatment 48 h and 24 h hours prior to delivery reduced the requirement for ventilator support and improved cardiovascular stability.

**Conclusion:**

The pig provides a relevant model for the study of human preterm physiology and for investigation of novel therapies to improve outcomes.

## Introduction

The preterm human neonate is subject to a number of acute diseases that are associated with a high risk of adverse outcomes. In the developed world, the incidence of preterm birth has increased over the last two decades but advances in neonatal care have reduced mortality [1]. Treatment with maternal glucocorticoids to accelerate fetal lung maturation and increase lung compliance [2], is now standard procedure and has been an important contributor to reductions in mortality and morbidity [3]. However the incidence of disability in survivors of preterm birth has not decreased.

Common serious clinical problems that preterm infants still face are respiratory distress syndrome [3], brain injury [4]_ENREF_1, cardiovascular instability [5], suboptimal growth [6] and necrotising enterocolitis (NEC) [7]. Adequate treatments have not yet been identified and those used in everyday practice have not been characterised nor fully tested. The development of novel therapies is hampered by deficiencies in knowledge of the relevant physiology. Increasing the knowledge of preterm physiology, and the evaluation of novel and existing clinical interventions, initially requires appropriate animal models.

An ideal animal model of human preterm physiology should produce viable neonates with maturation characteristics similar to a very preterm human infant, and have a body size that allows comparative monitoring, blood sampling and clinical interventions. Large animals such as sheep, dogs, pigs and cattle have been used to model adult physiology due to their large size and accessibility. The chronically cannulated sheep has been extensively used to study fetal physiology, particularly fetal hemodynamics, renal and lung function, endocrine function, sex differences and the effects of maternal glucocorticoid exposure. However the youngest use of a lamb *ex utero* is 125 days (0.85 of gestation) and survival was <3 h [8]. Below this gestation lung immaturity results in mortality, whereas many other organ systems are relatively mature and more representative of late preterm physiology [9].

The baboon model of the preterm neonate has been used extensively to study the impact of respiratory therapies [10]–[12]. Animals delivered at 125d (term approx. 185d gestation; 68%) are similar to a 26wk infant in that they require surfactant, ventilatory and hemodynamic support [12]. This non-human primate model has advantages because of the baboon’s similarity to the human. However the availability of these animals is very restricted and the cost is very high, limiting the feasibility of this model.

Research using the preterm pig has been more limited, with reported studies relating only to NEC in late preterm piglets. The youngest reported caesarean delivery of piglets is at 105d (0.91 gestation), by which time piglets are physiologically similar to a late preterm infant and do not require mechanical ventilation [13]. There is no information available regarding the viability and physiology of piglets at earlier gestations that may be equivalent to very preterm infants.

### Aim

The aim of this study was to describe the pig model of the preterm neonate in terms of its growth, physiology and requirement for intensive care over a range of gestational ages. In addition, the effect of maternal glucocorticoid treatment on some of these factors is described.

## Methods

### Ethics Statement

This study was carried out in strict accordance with the recommendations of the Australian Code of Practice for the Care and Use of Animals for Scientific Purposes (7th Edition). The protocol was approved by The University of Queensland Animal Ethics Committee (Approval numbers: UQCCR/998/08/NHMRC, UQCCR/999/08/NHMRC, PRC/684/07 and PRC/RBWH/687/07/NHMRC). All surgery was performed under anaesthesia and all efforts were made to minimize suffering.

### Animals

Artificially inseminated pregnant sows (Large white x Landrace) were sourced from a commercial piggery owned by The University of Queensland at Gatton, Australia. Piglets were delivered by Caesarean section at six gestational ages: 91d, 94d, 97d, 100d, 104d and 113d (term 115d) (Table 1). At each gestational age one to seven litters (11–73 piglets) were studied. Body weights and sex only were recorded at 3–24 h post-birth (postnatal age 1 day; PNA 1d) from 45 complete litters of pigs born by spontaneous vaginal delivery at term. Two additional groups of preterm piglets (at 91d and 97d) were exposed to maternally administered glucocorticoids (betamethasone, 0.19 mg/kg body wt, i.m.; Celestone Chronodose; Schering-Plough, USA) at 48 h and 24 h prior to delivery. This dose/kg is equivalent to that given to women presenting with threatened preterm labour.

**Table 1:** Gestational ages, sex ratios, body and organ weights of preterm piglets delivered by caesarean section.

Gestational age (days) Term = 115d	% gestation	No. Litters	No. Female and male piglets (F:M)	Body (g)	Heart (g)	Brain (g)	Liver (g)	Kidney (g)	Brain:liver
91	79	4	20∶20	697±193 (40)	5.2±1.3 (34)	18.6±1.7 (29)	16.4±4.5 (29)	2.9±0.8 (29)	1.20±0.31 (27)
94	82	1	5∶6	902±71 (11)	5.1±0.5 (5)	–	–	4.0±1.1 (5)	–
97	84	5	17∶23	881±213 (41)	7.0±1.6 (33)	21.9±2.2 (17)	19.0±4.3 (17)	4.3±1.3 (30)	1.20±0.29 (17)
100	87	2	13∶14	841±171 (27)	5.6±2.2 (18)	24.8±2.9 (21)	20.4±5.3 (18)	3.4±1.0 (18)	1.28±0.30 (18)
104	90	2	12∶12	1095±225 (24)	7.6±2.3 (15)	26.0±1.8 (15)	23.8±5.9 (15)	4.5±1.4 (15)	1.15±0.26 (15)
113	98	7	40∶33	1331±368 (73)	11.6±3.3 (53)	30.9±2.9 (25)	46.8±12.0 (20)	5.1±1.6 (49)	0.72±0.21 (19)
PNA 1d	100	45	-	1512±329 (460)	–	–	–	–	–
91+ GC	79	4	12∶24	715±149 (36)*	5.2±1.3 (31)	18.6±1.7 (20)	19.8±7.8 (27)	3.3±0.8 (27)	1.25±0.35 (20)
97+ GC	84	4	20∶27	1021±180 (46)	7.1±1. 3 (45)	23.5±2.0 (21)	35.9±6.2 (21)#	4.5±1.0 (36)	0.67±0.11 (20)

Weights are means ± SD (*n*). PNA (postnatal age) indicates piglets born at term by spontaneous vaginal delivery and weighed 3–24 h post-birth. GC indicates animals treated with maternal glucocorticoids prior to birth.

* *p* = 0.010, interaction effect between sex and maternal glucocorticoid treatment.

# *p*<0.001, effect of maternal glucocorticoid treatment.

### Caesarean Section

Pregnant sows (280–350 kg) were premedicated i.m. with 400 mg azaperone (Stresnil, i.m.; Janssen, Australia) and 1000 mg ketamine (approximately 3 mg/kg; Ketamil, i.m.; Troy Laboratories, Australia). An ear vein was catheterised for administration of anaesthesia which was induced with 200 mg of alfaxalone (approximately 0.6 mg/kg; Alfaxan-CD RTU, i.v.; Jurox, Australia), followed by intubation with a 14–16 mm endotracheal tube, with additional alfaxalone as required to allow intubation. The total administered dose of alfaxalone was 300–700 mg. Sows breathed spontaneously and were maintained with 2% isoflurane (Attane Isoflurane USP; Minrad, USA) in O_2_. Inhalational anaesthetic was used to facilitate rapid recovery of the piglets from the effects of maternal anaesthesia. Additionally, the i.v. drugs used to premedicate and induce anaesthesia will have minimal effects on piglet physiology as the dose is low and there is a substantial period of time (at least 2 hours) between dosing and commencement of piglet experimentation. Throughout surgery, saline (2–3L of 0.15 M NaCl) was administered via an ear vein and the following variables were monitored: arterial blood pressure by Doppler (Parks Medical Electronics Inc; Aloha, OR, USA), O_2_ saturation via pulse oximetry (Masimo; Masimo Corporation, Irvine, CA, USA), end tidal isoflurane and end tidal CO_2_ concentrations (Capnomac Anaesthesia Monitor; Datex-Ohmeda Inc, Madison, WI, USA). There was no significant hypotension or hypoxemia.

Caesarean delivery was performed via a ventral midline incision. Following incision into the linea alba the uterine horn was partially exposed. Complete exposure of the uterine horns was avoided so that uterine blood flow was not compromised by stretching or occlusion of the uterine arteries. Piglets were individually removed from the uterus at varying time intervals (up to 20 min) according to experimental requirements. Medication could be delivered to the fetus (including anaesthesia if required) by injecting into the umbilical vein prior to cord clamping. After all piglets were delivered, the sow was euthanased by i.v. injection of sodium pentobarbital (60 ml Lethabarb; Virbac, Australia).

### Piglet Resuscitation and Intensive Care

Where experimental protocols required piglet resuscitation, this was conducted in line with the current American Academy of Pediatrics (AAP) recommendations for human infants [14] but also took into account the requirements of the animal. Piglets were intubated and ventilated using a conventional neonatal ventilator and standard neonatal intensive care techniques. All ventilated piglets were sedated using a loading dose of fentanyl (5 µg/kg) and midazolam (0.2 mg/kg) followed by a maintenance infusion of fentanyl (2 µg/kg/h) and midazolam (6 µg/kg/h for <113d animals and 10 µg/kg/h for 113d animals). This protocol is similar to the current sedation protocol used in preterm infants [15]–[17]. Drug and replacement fluids (10% glucose at a rate of 3 mL/kg/h) were delivered via a 3.5FG dual lumen neonatal umbilical vein catheter (Argyle, Sherwood Medical, MO, USA).

### Physiological Monitoring

A 3.5FG neonatal umbilical artery catheter (Argyle, Sherwood Medical, MO, USA) was inserted into the umbilical artery for continuous invasive measurement of arterial blood pressure (Transpac® disposable pressure transducer; Hospira, USA). Blood was sampled intermittently for immediate blood gas analysis (ABL815 Blood Gas Analyser; Radiometer®, Denmark) and other analyses as required by experimental protocols. ECG was monitored using alligator clips attached to the skin of the thorax. Rectal temperature was maintained at 39.0°C±1°C (normal body temperature for pigs) using an overhead radiant heater (IW920 mobile infant warmer; Fisher & Paykel, New Zealand), with the addition of an under-bed heater mat for animals delivered before 100d gestation. Pulse oximetry was used to measure oxygen saturation (Radical; Masimo Corporation, Irvine, CA, USA). Blood pressure and oximetry data were continuously captured from the clinical monitors using a 16 channel Powerlab and Lab Chart v7 software (AD Instruments, Australia). ECG and temperature data were fed directly to the Powerlab.

Additional physiological measures and surgical procedures were possible in preterm piglets. The measurements described below were part of a specific experimental protocol and results of this study will be reported in a subsequent publication. Continuous single channel EEG was recorded using conventional EEG needle electrodes placed subcutaneously in the C3–C4 position (BRM2; BrainZ Instruments, New Zealand). Peripheral blood flow (skin blood flow) was monitored with a Laser Doppler flowmetry standard surface probe (AD Instruments, Australia) attached to the hind limb with an adhesive ring. Recording of ventricular pressure and injection of coloured microspheres to measure regional blood flow [18], [19] was achieved using a dual lumen catheter inserted into the left ventricle via carotid artery cut down. The position of the ventricular catheter was confirmed using the pressure waveform.

### Data Analyses

The effects of gestational age on body weight, relative organ weights (arcsine transformed) and brain:liver weight were detected using mixed model univariate ANOVA (where age was a fixed factor and litter was a nested random factor). At each age, the effect of sex on body weight and relative organ weights (arcsine transformed) was analysed using mixed model univariate ANOVA (sex was a fixed factor and litter was a nested random factor). The effect of maternal glucocorticoid treatment on body weight and relative organ weights (arcsine transformed) were analysed at 91d and 97d separately using univariate ANOVA (treatment and sex were fixed factors and litter was a nested random factor). The effect of gestational age, maternal glucocorticoid treatment and sex on hemodynamic and arterial blood gas parameters were analysed using univariate ANOVA (group and sex were fixed factors and litter was a nested random factor). For all analyses, significant differences are reported only where they existed independently of litter effects. Where an effect of group was detected Fisher’s least significant difference (LSD) tests were used to determine which groups were different. The effect of litter size on body weight was analysed separately for near term (113d and PNA 1d) and preterm (91d, 94d, 97d, 100d and 104d) pigs using a Spearman’s Rank correlation of body weight z scores and litter size. Reported values are means and SD unless stated otherwise. ANOVAs and correlations were conducted using SPSS Statistics version 20 (IBM, Chicago, IL, USA).

## Results

### Litters and Sex Ratios

A total of 29 litters of Large white × Landrace piglets (n = 298) were delivered by Caesarean section at six time points: 91d, 94d, 97d, 100d, 104d and 113d (term = 115d). Details of number of litters and piglets are included in Table 1. A further 45 litters spontaneously delivered at term were used to determine term weights. Litter size ranged from 5–17 animals (10.3±2.7, *n* = 74 litters). Litters were 53% male with the most extreme sex ratio 1∶9 (female:male) (Table 1). Data reported are for non-glucocorticoid treated animals unless stated otherwise.

### Growth

**Figure 1:**
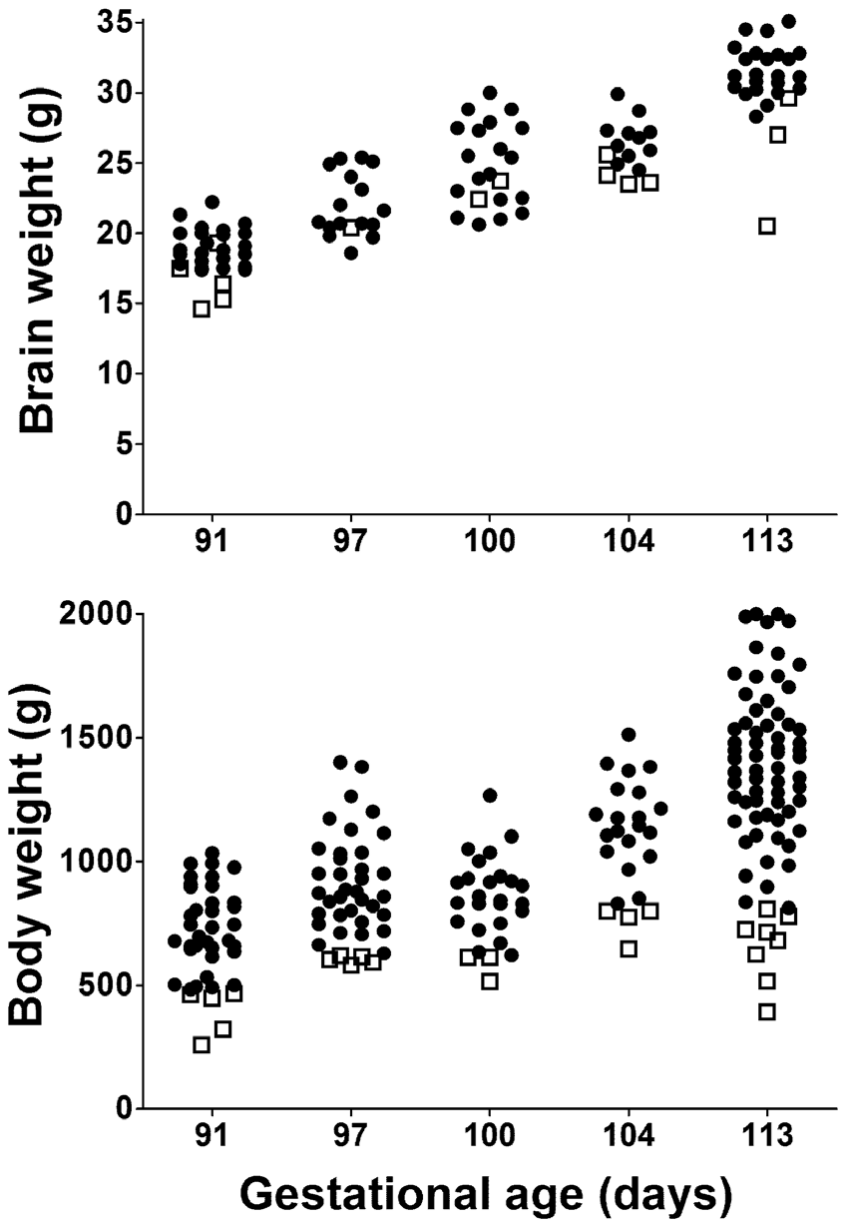
Brain and body weights of piglets over a range of gestational ages. Closed circles represent normal body weight animals and open squares represent animals with body weight <10^th^ centile. Not all piglets had brain weight recorded. Note the spread of weights within a given gestational age is greater for the body than for the brain implying that brain growth is relatively preserved even when body weight is low.

Body weight doubled between 91d and 113d (Table 1). Variability in body weight was high at all gestations as indicated by the high standard deviations (Table 1) and extensive scatter (Fig. 1), and this was the result of variation within each litter rather than large differences between litters. As a result there were often one or two animals in each litter with a birth weight close to the 10^th^ centile for the whole group and approximately half of litters also had one piglet with a body weight more than 40% below the mean for that litter. In contrast, there was less variation in brain weight with proportionally lower standard deviations (Table 1 and Fig. 1).

**Figure 2:**
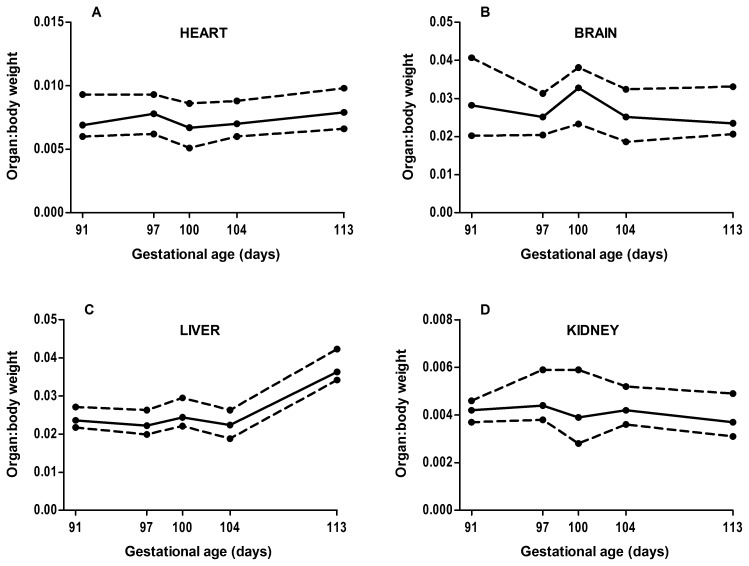
Relative organ growth from 91d to 113d of gestation (term = 115d) for A) heart, B) brain, C) liver, D) kidney. The solid line is the 50^th^ centile and the dashed lines are the 10^th^ and 90^th^ centiles. Note the weight of all organs increased proportionally with body weight, except for the liver which increased significantly relative to body weight between 104d and 113d (*p* = 0.004). Data from 94d was not included as only one litter was studied and few organ weights were recorded.

Males had significantly greater body weight compared with females at 91d (766±187 g *vs*. 627±177 g, *p* = 0.038) and 113d (1450±289 g *vs.* 1233 g±39 g, *p* = 0.019) only. Body weight was inversely correlated with litter size from 113d onwards (*r* = −0.236, *p*<0.01, *n* = 533) but not at earlier gestations (*r* = −0.023, *p* = 0.734, *n* = 225). The weight of the heart, brain and kidney relative to total weight did not increase from 91d to 113d (Fig. 2A, B, D). In contrast, the relative weight of the liver was constant from 91–104d, but had increased significantly between 104d and 113d (*p* = 0.004) (Fig. 2C). The brain:liver weight ratio was significantly higher (*p*<0.001) in animals born at less than 113d of gestation (1.13±0.35) compared with animals born at 113d (0.71±0.21). There was no significant effect of sex on relative organ weights at any gestational age.

### Physiology and Maturation

There was substantial variability, both within and between litters, in survival rates and requirements for additional ventilatory and cardiovascular support.

#### 113d gestation

These near term piglets appeared similar to a term animal with open eyes, good muscle tone and well developed skin and hair. Animals had adequate thermoregulation requiring minimal intervention to keep them warm. Respiration was spontaneous upon delivery and animals were hemodynamically stable. During experimental manipulation requiring general anaesthesia, intubation with a 3.0 cuffed endotracheal tube and ventilation at 15/5 cmH_2_O (peak inspiratory pressure/positive end expiratory pressure) at 30 breaths/minute in 21% O_2_ was generally adequate to maintain arterial pCO_2_ at 35–45 mmHg and oxygen saturation at 95% or above. Survival of these ventilated near term animals was 93% (28/30).

#### 104d gestation

At 104d, piglets had open eyes, good muscle tone and well developed skin and hair. Thermoregulation was poor, requiring external heat sources to maintain body temperature at 38–39°C. All animals breathed spontaneously.

#### 100d gestation

The eyelids of piglets at 100d gestation were partially fused, muscle tone was reduced, the skin was thin and some hair was present. Thermoregulation was poor and external heat sources were required. Most piglets autoresuscitated and breathed spontaneously and did not require mechanical ventilation. However in one litter, several animals exhibited intermittent apnea and required stimulation.

#### 97d gestation

At 97d piglets had fused eyelids, poor muscle tone and thin skin with minimal hair. Thermoregulation was very inadequate and it was difficult to maintain normothermia with overhead radiant heaters and heated mats. Hypothermia was reduced by delivery into a warm towel, drying the animal and then inserting it into a plastic bag that was open at either end. This reduced evaporative heat loss, allowed subsequent access to the head and umbilical cord, and is a practice used in the care of the very preterm human infant. All piglets required ventilation to survive. Prior to intubation, active suctioning of the oropharynx was required to remove lung liquid. Immediate intubation was then required with a size 2.0 mm uncuffed paediatric endotracheal tube (ETT) (Argyle, Tyco Healthcare Group, Mansfield, MA, USA), and administration of surfactant (4 ml/kg, Survanta, Abbott Laboratories, Columbus, Ohio USA) via the ETT was necessary to improve lung compliance and avoid death due to respiratory insufficiency or pneumothorax.

**Figure 3:**
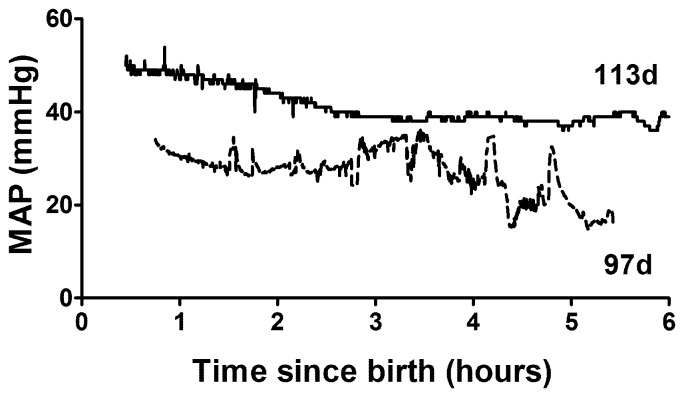
Mean arterial blood pressure (MAP) for a preterm (97d) and a term (113d) piglet (term = 115d) for six hours following delivery by Caesarean section. Note the preterm piglet has a lower and less stable mean arterial pressure (MAP).

Table 2 describes the physiology of piglets at 97d gestation compared to near term piglets (113d). Hemodynamic parameters, mean arterial pressure (MAP) and heart rate, are the average for a ten minute period occurring 30–40 min after resuscitation. MAP was lower and less stable compared with near term animals (mean 30 mmHg v. 36.5 mmHg; Table 2) (Fig. 3). Heart rate was similar in the two age groups. Arterial blood gas parameters from the first blood gas sample, typically taken 30–60 min post-birth, reflect the response to the initial ventilatory conditions rather than on-going maintenance. Ventilatory requirements were significantly higher for 97d animals than near term animals with peak inspiratory pressures of 25–30 cmH_2_O, respiratory rate of 60bpm and FiO_2_ up to 100%. However ventilatory requirements in these untreated preterm piglets were extremely variable and because some animals had poor saturation and very low pO_2_, the initial FiO_2_ was set quite high. In addition, during resuscitation some piglets exhibited symptoms consistent with pulmonary hypertension and so we decided to begin resuscitation with a high FiO_2_ in attempt to avoid this. As a result a high mean pO_2_ was recorded at the first blood gas with a very high SD (Table 2), but this was reduced to more normal levels as quickly as the piglets’ physiology allowed and was similar to term levels during experimentation (Table 2). Blood pH and arterial base excess (ABE) were also highly variable but on average piglets were more acidotic and had higher pCO_2_ compared to near term animals. At this gestational age, permissive hypercapnia (pCO_2_ of 45–55 mmHg) was used, but in the most immature piglets initial and sometimes ongoing pCO_2_ was above 70 mmHg. Pneumothorax, detected by clinical signs including chest asymmetry and reduced air entry, only occurred in one piglet and was secondary to severe respiratory failure requiring extremely high peak inspiratory pressures. The significant base excess appears normal for neonatal piglets and we have observed this in the many spontaneously delivered term piglets that we have studied over many years.

**Table 2:** Physiology of preterm and near term piglets.

Physiological Variable			Preterm (97 d)	Preterm+GC (97 d)	Near term (113 d)	P value
Mean arterial pressure (mmHg)		30.0±0.9 (15)*		29.2±0.8 (15)*	36.5±1.4 (14)	<0.001
Heart rate (bpm)		178±11 (15)		177±27 (15)	173±7 (16)	ns
pCO_2_ (mmHg)	initial	38.8±19.8 (17)		28.5±5.2 (16)	31.4±12.2 (16)	ns
	maintenance	36.4±9.0 (17)		41.7±7.0 (16)	35.8±2.8 (15)	ns
pO_2_ (mmHg)	initial	195±167 (17)		251±139 (16)*	121±51 (16)	0.025
	maintenance	147±71 (16)		112±36 (16)	111±32 (15)	ns
pH	initial	7.49±0.20 (17)		7.62±0.07 (16)* #	7.57±0.13 (16)	0.037
	maintenance	7.50±0.07 (17)*		7.46±0.07 (16)*	7.60±0.08 (16)	<0.001
ABE (mmol/L)	initial	3.7±3.9 (17)		7.1±2.4 (16)#	5.2±2.7 (16)	0.010
	maintenance	4.2±2.6 (16)		4.7±2.2 (16)	5.8±2.0 (15)	ns
FiO_2_ (%)	initial	96±9 (17)*		75±27 (16)* #	35±19 (16)	<0.001
	maintenance	70±26 (17)*		41±14 (16)#	32±20 (15)	<0.001
Hb (mmol/L)		81±23 (17)		89±9 (16)	74±19 (16)	ns

Physiological parameters of piglets delivered by caesarean section at 97 d gestation (untreated), 97 d (treated with maternal glucocorticoids = GC) and 113 d gestation (term is 115 d). Mean arterial pressure and heart rate have been averaged over a 10 min period (approximately 30 min post-birth). Initial arterial blood gas parameters are from the first blood gas taken 30–60 min post-birth and so reflect the response to the initial ventilatory settings. Maintenance values were obtained prior to experimentation and reflect ongoing maintenance. GC indicates animals treated with maternal glucocorticoids prior to birth. pCO_2_ is carbon dioxide partial pressure, pO_2_ is oxygen partial pressure, ABE is arterial base excess, FiO_2_ is fraction of inspired oxygen, Hb is haemoglobin. All values are mean ± SD (*n*).

* indicates significantly different to near term piglets (p<0.05).

# indicates a significant difference between untreated preterm piglets and preterm piglets treated with maternal glucocorticoids (p<0.05).

Difficulties were encountered with the measurement of pulse oximetry due to poor perfusion of the extremities. Survival was 83%, and 19 of the 23 resuscitated animals (not treated with maternal glucocorticoids) survived at least the 8 h required by the experimental protocol. There was no difference in survival between males and females.

#### 94 d and 91 d gestation

Piglets had very thin skin, poor muscle tone and no hair, and could not thermoregulate. Ventilation was extremely difficult even with the use of surfactant and high inspiratory pressures. Piglets were hemodynamically unstable and hypoxaemia was common. Pneumothorax occurred in 2 of 7 animals and specifically only in the context of severe respiratory failure which was being treated with extremely high peak inspiratory pressures. At 94 d, resuscitation was attempted on six piglets from one litter but all of these piglets survived <40 min primarily due to cardiovascular collapse and/or severe respiratory failure. At 91 d, resuscitation was attempted on only one piglet and it survived for <3 h due to cardiovascular collapse. At post-mortem, care was required when working with internal organs due to their delicate structure.

### Maternal Glucocorticoid Treatment

Delivery at 91 d gestation following a clinically relevant dose of maternal glucocorticoids, resulted in females with an increased body weight (799 g±107) compared with untreated females (627 g±177), and males (673 g±151) with a decreased body weight compared with untreated males (766 g±187) (*p* = 0.010). At 97 d there was no significant effect of treatment on body weight (*p* = 0.301). The relative weights of organs were not significantly affected by treatment at either 91 d or 97 d.

Ventilation of glucocorticoid exposed piglets was commenced at the same settings as for the untreated preterm piglets but FiO_2_ was rapidly reduced based on oxygen saturation. At the first blood gas, glucocorticoid treated animals already had a lower oxygen requirement (lower FiO_2_, Table 2). Nevertheless pO_2_ at the first blood gas was higher than in the untreated preterm piglets, pCO_2_ was lower and pH higher (Table 2) suggesting that further reductions were possible and inspiratory pressure could also be reduced. As in untreated preterm piglets ventilator settings were adjusted to obtain normal physiology as rapidly as possible (Table 2). Maintenance of glucocorticoid treated piglets was easier with a reduced requirement for higher peak inspiratory pressures (PIP) and FiO_2_, reduced time on 100% oxygen, and more stable cardiovascular parameters. In the glucocorticoid treated group 16 of 19 survived (84%) and survival was similar in males and females.

## Discussion

An ideal animal model of preterm physiology should produce viable neonates with maturation characteristics similar to preterm infants. We have demonstrated that with the application of standard neonatal intensive care techniques, preterm piglets at 97 d gestation can survive for at least 8 h.

From 97 d gestation, piglets’ lungs are sufficiently mature to allow successful resuscitation even without maternal glucocorticoid treatment. Piglets require similar levels of respiratory support to a human preterm infant and similar benefits of glucocorticoid exposure were seen [3]. Piglets delivered at 97 d have survived in our hands for the duration of experimental protocols lasting up to 8 h. To date we have not attempted to support longer survival but this is likely to be feasible as there is no major deterioration by 8 h of age.

The lower MAP and instability of blood pressure observed in the human preterm infant are also present in the preterm piglet [20], [21]. While there is a clear need to study the cardiovascular transition to *ex utero* life in the preterm baby there are few suitable models. The rat is born very immature and so makes this transition at an earlier developmental stage. The sheep heart is structurally more mature than the human infant as indicated by the proportion of binucleated myocytes being 70% [9] compared with about 10% in the human [22]. The preterm piglet may be the ideal model for the study of the preterm cardiovascular transitional period due to its similar physiology and also structural maturation (2–5% binucleated myocytes at 0.8 of gestation, increasing to approximately 10% at birth) (Kim MY, unpublished data).

We have not comprehensively evaluated brain development as the piglet has been previously validated as a superior model of normal human brain development. Specifically the timing of the perinatal brain growth spurt [23], patterns of cellular development and myelination [23], presence of gyri and sulci [24] are similar in piglets and human infants. In addition, understanding the pathophysiology of neonatal brain injury requires a model, such as the piglet, where the proportion of grey matter to white matter [24] and electroencephalography features [25] are more similar to human infants than that in lower order animals.

The larger size of piglets provides an advantage in physiological studies, enabling extensive monitoring and tissue and/or fluid sampling. Even at 91 d gestation, very preterm piglets are large enough to allow extensive continuous physiological monitoring including arterial blood pressure using umbilical arterial catheters, heart rate, pulse oximetry, respiration, ECG, ventricular pressure, EEG, peripheral blood flow and rectal temperature. Additionally, repeated blood and urine sampling is possible. This is not possible in small animal models.

Other advantages of the pig model are a consequence of the relatively large litter size. Firstly, large litters provide an opportunity for parallel experiments on interrelated questions to be answered in piglets from the same litters. Littermates can also be used as internal controls or to address sex differences. Secondly, pigs spontaneously produce growth restricted offspring. While many models of IUGR require surgical interventions to produce growth restricted fetuses [26], the presence of spontaneous growth restriction in piglets may provide advantages.

### Comparison to the Human Neonate

Varying developmental rates across species means it is not always appropriate to calculate equivalent gestational stages based simply on mathematics [27]. In addition, in rats and sheep some organs develop more rapidly than in the human infant while others develop more slowly [9], [28], [29]. In contrast, we and others have shown that the relative maturity of the lungs, cardiovascular system and brain [22]–[25], [29] of the piglet is comparable to that of the human infant. This is a major advantage of the piglet model.

A piglet born at 101–104 d of gestation requires minimal intervention beyond tactile stimulation and maintenance of body temperature, and is similar to a preterm infant >32 weeks gestation [30]. At 97 d of gestation, without maternal glucocorticoid treatment, piglets are physiologically similar to a very preterm baby born at 28–30 wk gestation, requiring surfactant for adequate ventilation and with some cardiovascular instability, but both have high survival rates [30]. At 91–94 d of gestation, ventilating and stabilising piglets without glucocorticoid exposure was extremely difficult and survival rates were low. These animals are the physiological similar to a preterm infant born at 23–25 wk of gestation [30].

### Maternal Glucocorticoid Treatment

Maternal glucocorticoid treatment plays a critical role in the management of preterm neonates so it is imperative that animal models demonstrate an appropriate response to treatment. Glucocorticoid treatment improved respiratory function with reduced requirement for higher PIP and FiO_2_, lower pCO_2_, and less acidosis at the first blood gas. Cardiovascular instability was also reduced. These positive effects of maternal glucocorticoid exposure on cardiopulmonary function in the preterm pig are similar to those observed in the human preterm neonate [31].

While no resuscitation was performed on 91–94 d piglets following maternal glucocorticoid treatment, improvements seen at 97 d suggest that survival rates at 91–94 d would be significantly increased by glucocorticoid exposure.

There is no clear consensus on the effects of clinically relevant doses of maternal glucocorticoid treatment on body and organ growth. There are no reported effects on fetal growth following a single course of treatment in humans [32], [33] however these studies combined the sexes possibly obscuring any opposing effects in male and females. Sheep studies using clinically relevant doses have shown conflicting results. One study found no effect on birth weight in a combined sex analysis [34], another observed reduced birth weight in both males and females with greater effects in females [35]. Another study showed a dose effect on growth in males only, with no effect on birth weight in females [36]. In the current study, exposure to clinically relevant doses of maternal glucocorticoids resulted in opposing sex specific effects on body weight. The weight of females increased and that of males decreased, but only for animals born at 91 d gestation. This surprising result was observed within every litter and remained statistically significant after the exclusion of outliers. This effect was not present when treatment was delayed by 6 d, suggesting that the sensitivity of growth to glucocorticoid administration may vary across gestation.

### Growth Restriction

The current study did not aim to identify pathogenetic issues associated with intrauterine growth restriction as this has already been extensively discussed [37]–[40]. However a number of observations were made that may assist others in the study of this important area. In term or near-term piglets we observed a significant correlation between piglet body weight and litter size, as previously reported by Bauer [41]. However this relationship was absent at earlier gestations suggesting nutrient supply to a large litter may not become a growth limiting factor for the whole litter until late in gestation. Despite this observation half of preterm litters did include a piglet with a body weight more than 40% below the mean for that litter. This may be the result of piglets developing in a section of the uterus where a borderzone of vascular supply exists leading to altered placental development [41].

Despite considerable variation in body weight within litters, variability in brain weight was low suggesting sparing of the brain in growth restricted piglets as previously reported [41]. At term the normal brain:liver weight ratio of piglets that have a body weight above the 10^th^ centile, is less than 1 [41] and a brain:liver weight of greater than 1 has been used to identify growth restriction. However, due to the rapid growth of the liver late in gestation, the normal brain:liver weight of piglets born at 104 d or earlier is greater than 1. Therefore, if this ratio is used to identify preterm piglets that are growth restricted a higher cut-off value will be required, perhaps 1.5.

### Suggested Improvements in the Model

There was extensive variability in how quickly piglets oxygenated so there is a need for rigorous early monitoring and adjustment of ventilation. In addition, the difficulties in ventilation and incidence of pneumothorax found in the untreated preterm piglets may be reduced by increasing the positive end expiratory pressure (PEEP) to 6–7 cmH_2_O. The glucocorticoid treated preterm piglets are less variable with reduced requirements for high FiO_2_ and high inspiratory pressure. To avoid hyperventilation in this group, initial ventilatory settings should be further adapted by reducing the PIP to 18–20 cmH_2_O, reducing the fraction of inspired oxygen (FiO_2_) to 60–70% and increasing the positive end expiratory pressure (PEEP) to 6–7 cmH_2_O.

### Limitations and Cautions

The major limitations of the preterm pig model are associated with the large size of the sow (350 kg) and requirement for equipment suitable for handling such weights. Intubation of sows is technically difficult. A large number of expert staff, including an experienced neonatologist, is required to deal with resuscitation and initiation of intensive care for the large numbers of piglets at preterm Caesarean section. A further limitation of the model is the high PIP initially required to adequately ventilate preterm piglets. The sedation used may alter piglet physiology. However maternal intravenous sedation is administered at very low doses and only very early in the procedure so that maternal levels should be extremely low by the time the piglets are delivered. Sedation administered to the piglets is designed to reflect NICU practices so that the physiology observed will more closely align with that of a preterm baby. Despite these limitations the preterm piglet provides an animal model that more closely resembles the human preterm neonate than other models.

### Conclusions

The pig provides a clinically relevant model of preterm neonatal physiology where the maturation of multiple organ systems is similar to the human early preterm infant. It can be used to test potential new treatments for major clinical problems such as respiratory distress syndrome, brain injury, cardiovascular compromise and NEC. Importantly this animal model provides the opportunity to explore major gaps in our understanding of the physiology of the preterm neonate. This knowledge is essential to the development of effective new therapies.
